# Metatranscriptomic evidence for classical and RuBisCO-mediated CO_2_ reduction to methane facilitated by direct interspecies electron transfer in a methanogenic system

**DOI:** 10.1038/s41598-019-40830-0

**Published:** 2019-03-11

**Authors:** Peixian Yang, Giin-Yu Amy Tan, Muhammad Aslam, Jeonghwan Kim, Po-Heng Lee

**Affiliations:** 1 Department of Civil and Environmental Engineering, Hong Kong Polytechnic University, Hung Hom, Kowloon, Hong Kong SAR, 999077 P. R. China; 2 Department of Environmental Engineering, Inha University, Inharo-100, Michuholgu, Incheon, 22212 Republic of Korea; 3 Department of Chemical Engineering, COMSATS University Islamabad (CUI), Lahore Campus, Defense Road, Off Raiwind Road, Lahore, 53720 Pakistan; 4 Dept. of Civil and Environmental Eng., Imperial College London, Imperial College Road, London, SW7 2BU UK

## Abstract

In a staged anaerobic fluidized-bed ceramic membrane bioreactor, metagenomic and metatranscriptomic analyses were performed to decipher the microbial interactions on the granular activated carbon. Metagenome bins, representing the predominating microbes in the bioreactor: syntrophic propionate-oxidizing bacteria (SPOB), acetoclastic *Methanothrix concilii*, and exoelectrogenic *Geobacter lovleyi*, were successfully recovered for the reconstruction and analysis of metabolic pathways involved in the transformation of fatty acids to methane. In particular, SPOB degraded propionate into acetate, which was further converted into methane and CO_2_ by *M. concilii* via the acetoclastic methanogenesis. Concurrently, *G. lovleyi* oxidized acetate into CO_2_, releasing electrons into the extracellular environment. By accepting these electrons through direct interspecies electron transfer (DIET)*, M. concilii* was capable of performing CO_2_ reduction for further methane formation. Most notably, an alternative RuBisCO-mediated CO_2_ reduction (the reductive hexulose-phosphate (RHP) pathway) is transcriptionally-active in *M. concilii*. This RHP pathway enables *M. concilii* dominance and energy gain by carbon fixation and methanogenesis, respectively via a methyl-H_4_MPT intermediate, constituting the third methanogenesis route. The complete acetate reduction (2 mole methane formation/1 mole acetate consumption), coupling of acetoclastic methanogenesis and two CO_2_ reduction pathways, are thermodynamically favorable even under very low substrate condition (down to to 10^−5^ M level). Such tight interactions via both mediated and direct interspecies electron transfer (MIET and DIET), induced by the conductive GAC promote the overall efficiency of bioenergy processes.

## Introduction

Energy recovery in the form of methane with sewage is of great interest^1^. Methanogenesis is accomplished by the syntrophic microbial interactions to achieve interspecies electron transfer (IET). Traditionally, IET was thought to be accomplished by syntrophs and methanogens through diffusive carriers (e.g. acetate, hydrogen and formate). This form of mediated IET (MIET) is constrained by the physical distance between syntrophs and methanogens, and the diffusion rate of electron carriers, creating a metabolic bottleneck^2^. However, there is growing evidence of an alternative direct interspecies electron transfer (DIET), which could overcome this bottleneck and enhance methane production rate^3,4^. DIET occurs in the presence of exoelectrogens, which are capable of shuttling electrons exogenously to methanogens through conductive pili or surfaces^3,5^. It is the predominant mechanism responsible for electron exchange in natural methanogenic communities aggregates^6^ and methanogenic system supplemented with electrically conductive particles such as magnetite (Fe_3_O_4_)^4^.

Energy efficient anaerobic fluidized membrane bioreactor (AFMBR) systems, which use granular activated carbon (GAC) as the fluidized media, are developed to treat domestic wastewaters with high energy harvest^7–9^. Effluent produced by anaerobic fluidized bed bioreactor (AFBR) was treated further by anaerobic fluidized bed ceramic membrane bioreactor (AFCMBR), termed as staged, anaerobic fluidized bed ceramic membrane bioreactor (SAF-CMBR). In these setups, GAC addition was originally conceived to serve as a mechanical scouring agent along membrane for reducing membrane fouling and a carrier for microbial attachment. Recently, we examined the microbial community in a staged anaerobic fluidized-bed ceramic membrane bioreactor (SAF-CMBR) consisting of anaerobic fluidized-bed bioreactor (AFBR) followed by anaerobic fluidized-bed ceramic membrane bioreactor (AFCMBR), fed with acetate and propionate^8^. This presented an ideal system for investigating syntrophic microbial interactions as these substrates are the key precursors of methanogenesis, driving IET in energy-limited methanogenic systems^10–12^. We observed the co-dominance and tight interactions between syntrophic propionate oxidizing bacteria (SPOB), *Syntrophobacter and Smithella*, acetoclastic methanogen *Methanothrix*, and exoelectrogen *Geobacter* on the GAC particles, suggesting that GAC serves as an electrically conductive material for promoting DIET in the SAF-CMBR^8^. This lends further credence to the growing literature on GAC-facilitated DIET^3^. Nevertheless, the metabolic interactions, particularly between *Methanothrix* and *Geobacter*, have yet to be fully understood. Such energy efficient SAF-CMBR was an ideal system to explore the underlying metabolic mechanisms and to provide their exploitation for the further improvement of the reactor design and operation.

Competition may occur between *Methanothrix* and *Geobacter* as both species utilize acetate for methanogenesis and respiration^13,14^. However, synergetic interaction between these two microbes is also possible. Most interestingly, while *Methanothrix* is unable to perform hydrogentrophic methanogenesis using CO_2_ due to the absence of a hydrogen uptake mechanism^15^, it can form syntrophic association, termed ‘electric syntrophy’, with *Geobacter* to achieve methane production, in a similar fashion to hydrogentrophic methanogenesis, through DIET-dependent CO_2_ reduction^6,16^. On the other hand, Kono *et al*. (2017) recently proposed a ribulose-1,5-bisphosphate carboxylase/oxygenase (RuBisCO)-mediated CO_2_ fixation pathway in many methanogen species including *Methanothrix*. It could reduce CO_2_ into various carbon intermediates for important metabolic pathways, such as gluconeogenesis and glycolysis, with acetate or/and hydrogen Kono, Mehrotra, Endo, Kizu, Matusda, Kimura, Mizohata, Inoue, Hasunuma, Yokota, Matsumura and Ashida^17^. This newly-discovered pathway, termed “reductive hexulose-phosphate (RHP) pathway”, is analogous to the Calvin–Benson cycle in plant photosynthesis, raising our hypothesis that it could be electron-driven (i.e., DIET). Additionally, this carbon fixation pathway has a potential link with methanogenesis via a formaldehyde intermediate, leading to our speculation of a third methanogenesis route in *Methanothrix*.

This study examines the interspecies interactions, particularly between *Geobacter* and *Methanothrix*, on GAC surfaces in the propionate- and acetate-fed SAF-CMBR. By employing a combinatorial approach of metagenomics and metatranscriptomics sequencing, this current enquiry aims to disclose potential methane formation pathways, facilitated by MIET and DIET, and their metabolic link in *Methanothrix*. The efficient and stable operation of methanogenic bioreactors relies heavily on syntrophic-driven IET mechanisms. A deeper understanding of such interactions is therefore critical to ultimately tie ecology to improvements in engineering operation and design.

## Results and Discussion

### Overview of the Metagenome Bin and Metatranscriptomes

GAC microbial communities of both AFBR and AFCMBR in the SFA-CMBR were predominated by SPOB (*Syntrophobacter and Smithella*), acetoclastic methanogens (*Methanothrix*), and the exoelectrogen *Geobacter*^8^. To further examine the metabolic interactions between these species, high quality genome bins (with >90% completeness) for these microbes were recovered through metagenomic short reads and hybrid assemblies (Tables S2). Three high-quality genome bins, AFBR_GAC_Bin72, AFCMBR_GAC_MaxBin.090 and AFBR_GAC_MaxBin.001, were phylogenetically identified to be closely related to the genomes of *Syntrophobacter fumaroxidans*, *Methanothrix concilii* and *Geobacter lovleyi*, respectively (Fig. S2).

**Figure 1:**
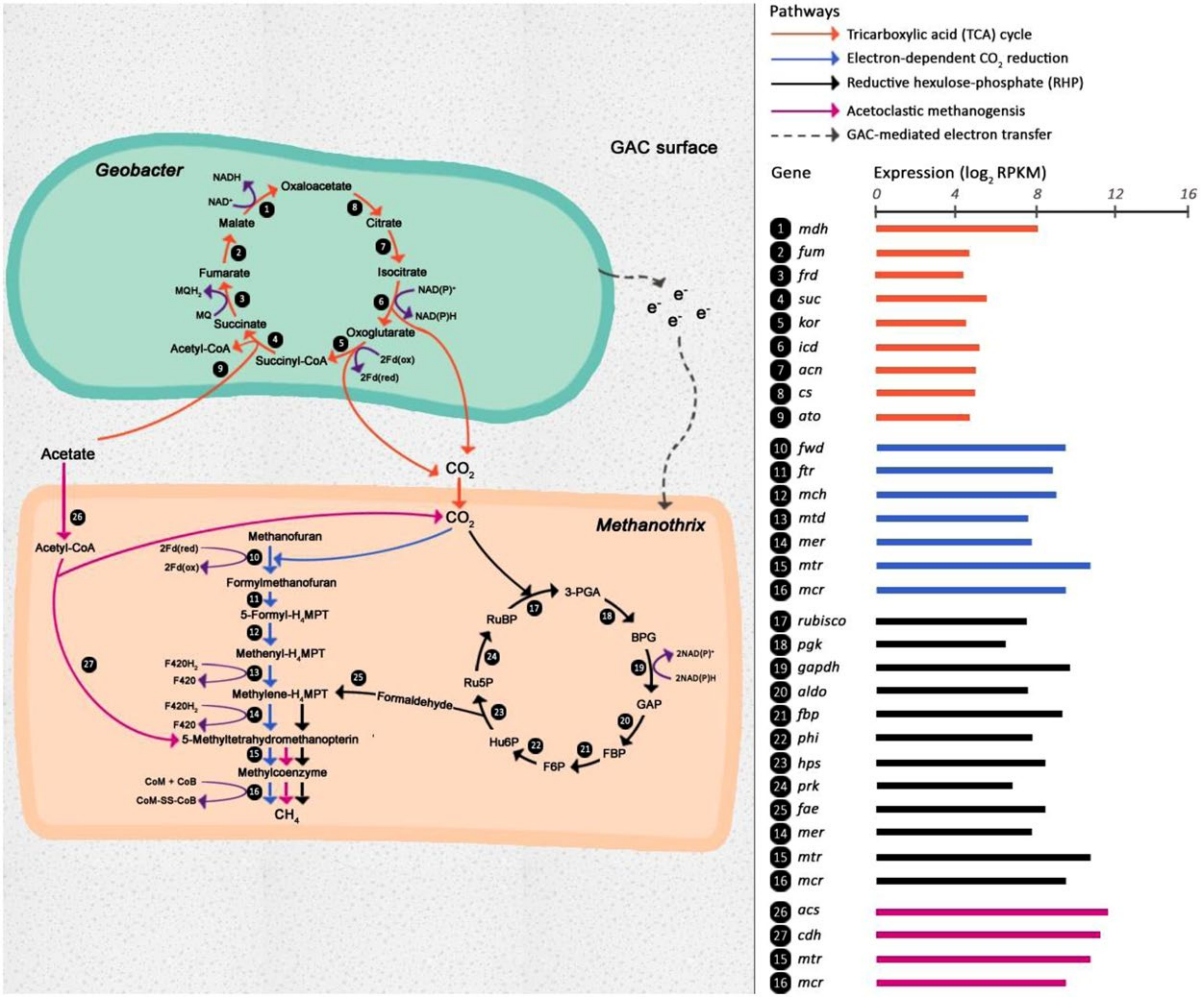
Annotated pathways of acetate oxidation, acetocalstic methanogenesis, classical CO_2_ reduction and CO_2_ reduction via RHP for methane production. Expression level of involved genes were evaluated as the log_2_ RPKM values and represented by the bar chart. Ru5P, ribulose-5-phosphate; RuBP, ribulose-1,5-bisphosphate; 3-PGA, 3-phosphoglycerate; BPG, 1,3-diphosphoglycerate; GAP, glyceraldehyde-3-phosphate; FBP, fructose-1,6-bisphosphate; F6P, fructose-6-phosphate; Hu6P, D-arabino-3-hexulose-6-phosphate; H4MPT, tetrahydromethanopterin.

The complete pathways for propionate degradation and acetate oxidation were recovered from the *S. fumaroxidans* MPOB (AFBR_GAC_Bin72) genome bin and *G. lovleyi* genome bin (AFBR_GAC_MaxBin.001), respectively. The acetoclastic methanogenesis, classical CO_2_ reduction and RHP pathways were also fully recovered from the *M. concilii* genome bin (AFCMBR_GAC_MaxBin.090) (Fig. 1; Table S2). Metatranscriptomics analysis confirmed that the genes involved in the aforementioned pathways were actively-transcribed.

### Propionate oxidation and acetoclastic methanogenesis

In our propionate and acetate-fed methanogenic system, propionate was first degraded into acetate and hydrogen, thereby acetate was adequately available as electron donor for further metabolization. *Syntrophobacter* degrade propionate into acetate using methylmalonyl-CoA (MMC) pathway adopted by most SPOB^11^. While a unique syntrophic propionate oxidizer, *Smithella* was transcriptionally-active and it degrades propionate into acetate via butyrate using a distinctly different pathway, of which the genes involved are still unclear so far^18^. On the other hand, the MMC pathway was fully reconstructed in the *S. fumaroxidans* MPOB genome bin, and these genes were found to be highly expressed with log_2_ RPKM values of 5.17–10.13 (Tables S3, S4). Therefore, such acetate-rich environment was favorable for acetate-utilizing microbes, explaining the enrichment of exoelectrogenic *Geobacter* and methanogenic *Methanothrix*. Moreover, *G. lovleyi* is capable to use hydrogen as an alternate electron donor and exhibits lower hydrogen consumption threshold concentration than that of methanogen^14^. The limited hydrogen produced from the propionate degradation pathways was too low to favor the growth of hydrogenotrophic methanogens in the system, while it could be utilized by *G. lovleyi* and further facilitated its dominance. This was reflected by the observation that the abundance of hydrogenotrophic methanogens was relatively low and *G. lovleyi* dominated in the community.

Acetate, which acts as an electron diffusive carrier of MIET, is assimilated by acetoclastic methanogens for methane generation. The acetoclastic methanogenesis pathway, which splits acetate into a methyl group and an enzyme-bound CO then further reduced to methane^19^, was fully reconstructed in the *M. concilii* genome bin (Table S3). The genes involved were also found to be highly expressed in the genome bin (log_2_ RPKM values of 5.45–11.96) (Table S4; Fig. 1), indicating that *M. concilii* was metabolically active and contributed to the methane production via its acetoclastic methanogenesis pathway.

In propionate-fed syntrophic community, the metabolic activities of SPOB and methanogens are intimately dependent on each other^20^. Collectively, these findings confirmed that a syntrophic interaction was present between GAC-dwelling acetoclastic methanogen *M. concilii* and SPOB, *S. fumaroxidans* MPOB and *Smithella. sp*., to achieve the complete bioconversion of propionate to CH_4_ and CO_2_. Given that these microbes were selectively enriched on the GAC in the SFA-CMBR^8^, it is most likely due to GAC facilitated MIET by enabling the microbes to grow in proximity of each other.

### Acetate oxidation and DIET-dependent CO_2_ reduction

Pathway reconstruction in the draft genome bin related to *G. lovleyi* showed the capability of acetate utilization and CO_2_ production via the tricarboxylic acid (TCA) cycle (Fig. 1; Table S3). Gene expression patterns further confirmed that the acetate oxidation pathway was metabolically active in *G. lovleyi* (log_2_ RPKM values of 4.53–8.30) (Fig. 1; Table S4). The high abundance of gene transcripts was also observed when two *Geobacter* species were co-cultured together and acetate was available as an electron donor^5^. Both the *G. lovleyi* acetate oxidation and *Methanothrix* acetoclastic methanogenesis pathway were found to be transcriptionally-active, indicating that they were competing for acetate at the substrate-level. Concurrently and more importantly, they also formed an electric syntrophic relationship and benefited each other via the IET. It was reported that the growth of *Geobacter spp*. was suppressed when methanogenesis was inhibited, suggesting that *Geobacter* grew under syntrophic or synergetic association with methanogens^16^. Methanogens are possibly “electron receivers” and serve as electron sinks of the dissipated electrons from *Geobacter*.

Why such a substrate-competing relationship exists within a cooperative association? Is it associated with DIET-induced interactions attributed to the electrically-conductive GAC? Indeed, acetoclastic methanogenesis was not the only transcriptionally-active pathway detected in the *M. concilii* genome bin. The DIET-dependent CO_2_ reduction methanogenesis pathway was also recovered (Fig. 1; Table S3). The genes specifically associated with the CO_2_ reduction pathway (*fwd*, *ftr*, *mch*, *mtd* and *mer*) were highly expressed at levels close to the acetoclastic methanogenesis pathway (log_2_ RPKM values of 5.89–9.68) (Fig. 1; Table S4). Unlike hydrogenotrophic methanogens, *Methanothrix* is incapable of performing CO_2_ reduction to methane via MIET as it cannot uptake reducing equivalents in the form of hydrogen and formate^5,19,21^, suggesting that DIET-driven methanogenesis was prevalent within the GAC community of SFA-CMBR. This observation agrees with a finding that *Geobacter* species could transfer electrons to *Methanothrix* species to support CO_2_ reduction via DIET^22,23^. In other words, *G. lovleyi* and *M. concilii* also established a close “electric syntrophic” relationship for the generation of methane from CO_2_. The sources of CO_2_ could be extracellular (CO_2_ released from propionate oxidation and TCA cycle in SPOB and *Geobacter*, respectively) and intracellular (CO_2_ as a byproduct from acetoclastic methanogenesis). In *M. concilii*, the by-product of MIET facilitated pathway (acetoclastic methanogenesis), CO_2_, was further utilized in the DIET facilitated pathways (CO_2_ reduction). By coupling the MIET and DIET, *M. concilii* could utilize the metabolite, CO_2_, for additional energy capture. Accordingly, in this SFA-CMBR, such interspecies interactions facilitated DIET-dependent pathway and promoted the overall energy recovery in the form of methane.

### CO_2_ reduction via the RHP pathway

Besides the classical CO_2_ reduction, the RHP pathway for carbon fixation is expected to be widely distributed in methanogenic archaea and the genes in such pathway are conserved in *M. concilii*^17^. Indeed, the complete RHP pathway was identified in the *M. concilii* genome bin (Fig. 1; Table S3). All the genes involved in the RHP pathway were at equally high expression levels as the acetoclastic and classical DIET-dependent CO_2_ methanogenesis pathways with log_2_ RPKM values ranging between 6.05 and 9.79 (Fig. 1; Table S3). This provides the first definitive proof that the entire RHP pathway is metabolically active in *M. concilii*. The RHP pathway, similar to the Calvin–Benson cycle, includes three phases: carbon fixation, carbon reduction, and ribulose-1,5-bisphosphate (RuBP) regeneration^17^. A study analyzing the RHP pathway *in vivo* for *Methanospirillum hungatei* showed that a small proportion of carbons fixed by RuBisCO were recycled for RuBP regeneration in the RHP pathway, and while a high proportion of fixed carbons were supplied to gluconeogenesis and glycolysis^17^. Also, it was proposed that the archaea invested a much smaller fraction of energy in the RHP pathway as compared to plant’s energy investment in the Calvin–Benson cycle^17^. By accomplishing this carbon fixation pathway with relatively low-energy investment, *M. concilii* could achieve further cell synthesis, therefore facilitating the dominance of *M. concilii* in the community and strengthened their overall activities. Hence, it is very likely that the RHP pathway plays an important role in *M. concilii* anabolism.

An important question is if the RHP pathway in *M. concilii* mediates methane production. The formaldehyde intermediate has been speculated to act as a metabolic link between the RHP pathway and methanogenesis in methanogens^17^. Accordingly, formaldehyde released from the RHP cycle can be condensed with tetrahydromethanopterin to form methyl-H_4_MPT, which is a key methanogenic precursor also central to both the methanogenesis and classical CO_2_ reduction pathways (Fig. 1). Four copies of the 5,6,7,8-tetrahydromethanopterin hydrolyase gene (*fae*), which perform formaldehyde condensation, were successfully recovered from the *M. concilii* genome bin (Fig. 1; Table S3). The high expression levels of *fae* (log_2_ RPKM values of 7.54–8.66) strongly suggest the involvement of the RHP pathway in methanogenesis. Moreover, similar to the classical DIET-dependent CO_2_ methanogenesis, the RHP carbon fixation pathway is also an electron-consuming process. This raises the possibility that the RHP pathway could be relying on external electrons received from *G. lovleyi* through DIET.

### Thermodynamics estimation of the CO_2_ reduction pathways

The transcriptional activity of all MIET and DIET-facilitated methanogenesis pathways meant that they were all concurrently happening. Therefore, the thermodynamics of each pathway was explored. Table 1 summarizes the reactions of acetoclastic methanogenesis (Eq. 1), CO_2_ reduction to methane (Eq. 2), CO_2_ fixation via RHP pathway (Eq. 3). At biological conditions (298 K and pH 7.0), the standard Gibbs free energy changes (∆G^0′^) of Eq. 1–Eq. 3 were calculated (Table S5). Given that the intracellular-produced CO_2_ from acetoclastic methanogenesis could serve as a substrate for CO_2_ reduction in *M. concilii*, a concurrent MIET and DIET activity could result in a complete acetate reduction to methane (2 mole of methane formation per 1 mole acetate consumption). This is reflected in the summation of acetoclastic methanogenesis with classical CO_2_ reduction (Eq. 4) and RHP pathway (Eq. 5). As shown in Table 1, all these discussed reactions were thermodynamically favorable under standard biological conditions since all the ∆G^0′^ values were far below zero. Additionally, the energy released/yielded from the classical CO_2_ reduction (−86.95 kJ mol^−1^) and RHP pathway (−53.95 kJ mol^−1^) are significant and higher than that from acetoclastic methanogenesis, when there is an incoming electron supply for *M. concilii* through DIET. With facilitation of DIET, methanogens can achieve CO_2_ reduction and yield more energy compared to conditions without external electrons. The yielded energy in *M. concilii* results in more methane formation, therefore improving the overall energy recovery efficiency of the AFCMBR.

**Table 1:** Reactions of methane production in the system and the corresponded standard Gibbs free energy.

Number	Description of reaction	Reaction	∆G^0′^ (kJ mol^−1^)
Eq. 1	Acetate methanogenesis	CH_3_COOH (aq) → CH_4_ (aq) + CO_2_ (aq)	−24.84
Eq. 2	Classical CO_2_ reduction	CO_2_ (aq) + 2 Fd (red) + 2 F_420_ (red) + CoM-SH + CoB-SH → CH_4_ (aq) + 2 Fd (ox) + 2 F_420_ (ox) + CoM-SS-CoB + 2H_2_O	−62.11
Eq. 3	CO_2_ reduction via the RHP pathway	CO_2_ (aq) + 2 NADP (red) + F_420_ (red) + CoM-SH + CoB-SH → CH_4_ (aq) + 2 NADP (ox) + F_420_ (ox) + CoM-SS-CoB + 2H_2_O	−29.11
Eq. 4	Complete acetate reduction via classical CO_2_ reduction	CH_3_COOH (aq) + 2 Fd (red) + 2 F_420_(red) + CoM-SH + CoB-SH → 2 CH_4_ (aq) + 2 Fd (ox) + 2 F_420_ (ox) + CoM-SS-CoB + 2H_2_O	−86.95
Eq. 5	Complete acetate reduction via the RHP pathway	CH_3_COOH (aq) + 2 NADP (red) + F_420_ (red) + CoM-SH + CoB-SH → 2 CH_4_ (aq) + 2 NADP (ox) + F_420_ (ox) + CoM-SS-CoB + 2H_2_O	−53.95

The standard free energy change (∆G^0′^) was calculated from the standard free energies of formation at 298 K, pH at 7.0 with CO_2_ and CH_4_ in the aqueous state and all compounds at 1 molar activity.

**Figure 2:**
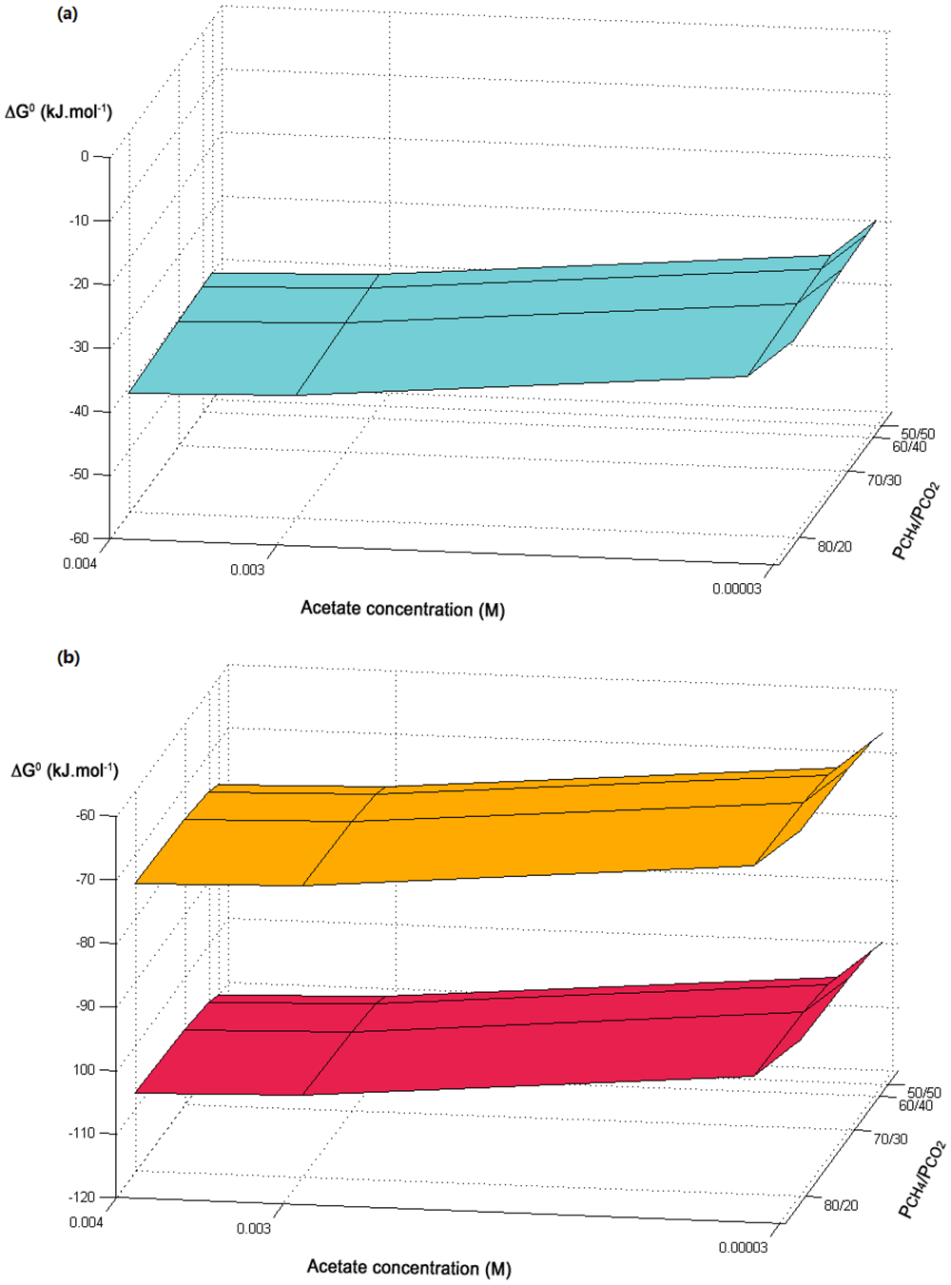
Transformed Gibbs free energy values (∆G^′^_298K_) in kJ mol^−1^ at pH of 7 and pressure of 1 atm as a function of acetate concentration and partial pressure ratio of CH_4_/CO_2_ for (**a**) Eq. 1: acetoclastic methanogenesis (**b**) Eq. 4 (complete acetate reduction via classical CO_2_ reduction) and Eq. 5 (complete acetate reduction via RHP pathway). The red curve represents Eq. 4, and the red orange one represents Eq. 5.

To further evaluate thermodynamic feasibility of these reactions in the AFCMBR, the transformed Gibbs free energy values (∆G′) at 298 K and pH 7 were estimated within an acetate concentration range of 0.03 mM–4 mM and a CH_4_/CO_2_ partial pressure ratio of 1–4, which mimics the actual conditions prevalent in the AFCMBR and other anaerobic digestion systems. Figure 2a displays the variation of ∆G′ for acetoclastic methanogenesis, indicating that the reaction can proceed even under very low acetate concentration. The energy gain from acetoclastic methanogenesis gently decreases as the acetate concentration decreases, while its effects on ∆G′ became more obvious at extremely low acetate concentration. In comparison, within the set range, changes of partial pressure ratio of CH_4_ to CO_2_ exerted insignificant influence on the energy gain. On the other hand, as shown in Fig. 2b, both the complete acetate reduction reactions were highly driven, and both of their energy gains (−60 to −80 and −90 to −110 kJ mol^−1^) were higher than that of acetoclastic methanogenesis alone without further CO_2_ reduction (−30 to −40 kJ mol^−1^). Therefore, when there are electrons available via DIET for *M. concilii*, the thermodynamic driving force for further CO_2_ reduction and/or the complete acetate reduction into methane is favorable. Notably, the energy gain from the complete acetate reaction via classical CO_2_ reduction is higher compared to the one via the RHP pathway, due to the differences of each metabolism involved intrinsically. Similar to the acetoclastic methanogenesis, ∆G′ of the two complete acetate reduction reactions were hardly affected by the partial pressure ratio of CH_4_/CO_2_, and their energy gains gently enhances as the acetate concentration increases. Overall, all the three reactions were thermodynamically feasible under this SFA-CMBR even at very low substrate concentrations. The thermodynamics calculation verified the feasibility of the acetoclastic methanogenesis and classical and RHP CO_2_ reduction pathway under such system conditions. With these two CO_2_ reduction pathways facilitated by DIET, the energy gain from the complete acetate reduction was higher that acetoclastic methanogenesis alone without further CO_2_ reduction.

## Methods

### Sample collection

The fluidized GAC samples were taken from the propionate- and acetate-fed (250 mg COD L^−1^) SAF-CMBR on Day 162, when the system was at steady state, for metagenomics and transcriptomics sequencing^8^. Genomic DNA extraction method was described in the previous study^8^. Shotgun metagenomic library construction and sequencing was performed on Illumina HiSeq. 4000 platform (Illumina, San Diego, CA, USA) at BGI (Shenzhen, China), generating paired-end (PE) reads with a read length of 150 base pair (bp). About 50 Gbp of metagenomic data per sample was generated. Meanwhile, 10 kb metagenomic library construction and sequencing were also performed on the PacBio Sequel Platform (Pacific Biosciences of California, Menlo Park, CA). Single-end (SE) reads with a mean insert length of 5700 base pair (bp), and 11.87 Gbp (AFBR_GAC) and 5.34 Gbp (AFCMBR_GAC) of metagenomic data were generated.

The two GAC samples were obtained from AFBR and AFCMBR for metatranscriptomics sequencing when CH_4_ generation reached steady phase. Experimental conditions of SAF-CMBR system are explained detail in our previous work^8^. The samples were preserved with *RNAlater* solution (Thermofisher, USA) in a volume ratio of 1:1 immediately after sample collection and frozen at −20 °C overnight. Subsequently, the samples were sent to BGI (Shenzhen, China) for total RNA extraction, metatranscriptomic library construction and metatranscriptomic sequencing on Illumina Hiseq. 4000 platform (Illumina, San Diego, CA, USA). rRNA was removed with kit after total RNA was collected from. Fragmentation buffer was added for interrupting mRNA to short fragments. Paired-end (PE) with read length of 150 bp and 15.4Gbp of metatranscriptomics data per sample was generated.

### Genomic analysis

For metagenomics analysis, the raw reads were first trimmed with a minimum quality cutoff of 3, and further screened to be at least 78 bp in length, having an average quality score >30 and containing less than 3 ambiguous nucleotides (N’s) using Trimmomatic^24^. Followig which, digital normalization was performed to remove redundant sequences with khmer scripts (k-mer size 20). Paired-end reads were *de novo* assembled into long sequence contigs using St. Petersburg genome assembler (SPAdes, version 3.9.0) based on de Bruijn graph with default settings (“-k 19,33,47,61,75–careful”)^25,26^. Meanwhile, a parallel hybrid assembly was performed on the trimmed reads obtained from the Illumina HiSeq platform together with the trimmed reads obtained from the PacBio Sequel Platform using the same parameters. Next, MaxBin was used for binning the assembled contigs into taxonomic bins based on an Expectation-Maximization algorithm^27^. Then, CheckM was performed to assess the quality of draft genomes using a broader set of marker genes specific to the position of a genome within a reference genome tree and information about the collocation of these genes^28^. The recovered genome bins were phylogenetically identified by comparing with reference genomes using PhyloPhlAn^29^. Prokka (version 1.11)^30^ was used annotated protein coding genes. Then gene functions were further characterized and functional pathways were reconstructed with BlastKOALA^31^.

For metatranscriptomics analysis, the raw reads were first trimmed with a minimum quality cutoff of 3, and further screened to be at least 50 bp in length, having an average quality score >30 and containing less than 3 ambiguous nucleotides (N’s) using trimmomatic^24^. Assembly was performed on the paired-end trimmed reads using Trinity^32^. Afterward, Sequence Expression AnaLyzer (Seal) in the BBTools suite was used to map the assembled metatranscriptomic file against the recovered high-quality genome bins generated from metagenomic analysis pipeline under “ambig modes”^33^. Genes expression level was evaluated based on generated Reads Per Kilobase Million (RPKM) and calculated as log_2_ RPKM values.

## Supplementary information

Supplementary information
