# Supplementary material for: Metatranscriptomic evidence for classical and RuBisCO-mediated CO2 reduction to methane facilitated by direct interspecies electron transfer in a methanogenic system
Source: Sci Rep. 2019 Mar 11;9:4116. doi: 10.1038/s41598-019-40830-0 (PMC6411985; doi:10.1038/s41598-019-40830-0)
Supplement: Supplementary file 1 — Supplementary information [file 41598_2019_40830_MOESM1_ESM.docx]

**Supplementary Information to “Metatranscriptomic evidence for classical and RuBisCO-mediated CO2 reduction to methane facilitated by direct interspecies electron transfer in a methanogenic system”**

Peixian Yang^1^, Giin-Yu Amy Tan^1^, Muhammad Aslam^2, 3^, Jeonghwan Kim^2^, Po-Heng Lee^1*^

*1 Department of Civil and Environmental Engineering, Hong Kong Polytechnic University, Hung Hom, Kowloon, Hong Kong SAR, P. R. China.*

*2 Department of Environmental Engineering, Inha University, Namgu, 100 Inha-ro, Incheon, Korea*

*3 Department of Chemical Engineering, COMSATS Institute of Information Technology, Lahore, Pakistan*

^*^*Corresponding author:* *phlee@polyu.edu.hk; phone: +852-2766-6067; fax: +852 2334 6389*

**Number of pages: 26**

**Number of tables: 5**

**Number of figures: 2**

1. **Genome reconstruction and metabolic pathways characterization by metagenomic analysis**

In total, around 313.8 million of 150 bp reads were obtained for the samples of AFBR GAC and AFCMBR GAC by paired-end sequencing on Illumina HiSeq platform (Table S1). Following read quality control and *de novo* assembly, a total of 543293 and 655739 contigs with N50 of 1890 bp and 1707 bp were retained for AFBR GAC and AFCMBR GAC, respectively (Table S1). Following by binning of the metagenomic sequencing reads, 56 (23 and 33) of draft genomes with high-quality (estimated > 90% completeness) were recovered from AFBR GAC and AFCMBR GAC. To further improve the quality of recovered genome bins, hybrid assembly was performed with the trimmed paired-end (PE) reads sequenced on Illumina HiSeq platform (Illumina, San Diego, CA, USA) and the trimmed single-end (SE) reads sequenced on the PacBio Sequel Platform. *de novo* assembly and Maxbin binning yielded 85 (37 and 48) of genome bins with high completeness in AFBR_GAC and AFCMBR_GAC. These genome bins were phylogenetically identified by genomic comparison using PhyloPhlAn. and the genomes closely related to syntrophs, methanogens and *Geobacter* were further analyzed (Table S2). These recovered genome bins are highly consistent with the abundant OTUs of the 16S rRNA gene analysis ^1^. Specifically, three high-quality bins with relatively high completeness, low contamination and low heterogeneity (Fig S1) were further annotated for pathways reconstruction, and the bins AFBR_GAC_Bin72, AFCMBR_GAC_MaxBin.090 and AFBR_GAC_MaxBin.001 were closely related to the genomes of *Syntrophobacter fumaroxidans* MPOB*, Methanothrix concilii* GP 6 and *Geobacter lovleyi* SZ, respectively (Fig S2). These recovered genome bins were highly consistent with the abundant OTUs of the 16S rRNA gene analysis. The gene content of these reconstructed genome bins was annotated (Table S3), thereby illuminating the potential functional properties of the microbial system.

1. **Experiment pipeline of prokaryotic strand-specific transcriptomes**

rRNA was removed with kit after total RNA was collected from prokaryote. Fragmentation buffer was added for interrupting mRNA to short fragments. Taking these short fragments as templates, Random hexamer-primer were used to synthesize the first-strand cDNA. The second-strand cDNA was synthesized using buffer, dATPs, dGTPs, dCTPs, dUTPs, RNase H and DNA polymerase I respectively after removing dNTPs. Short fragments were purified with QiaQuick PCR extraction kit and resolved with EB buffer for end reparation and adding poly(A). After that, the short fragments were connected with sequencing adapters. Subsequently, the UNG enzyme was used to degrade the second-strand cDNA, and the product was purified by MiniElute PCR Purification Kit before PCR amplification. The library was sequencing using Illumina HiSeq4000 (Illumina, San Diego, CA, USA).

1. **Evaluation of genes expression in the metabolic pathways of interests by metatranscriptomic analysis**

After trimming and assembly of metatranscriptomic sequencing reads, the output fasta file was inputted for mapping against the recovered genomes bins of interests. Specifically, the genome bins AFBR_GAC_Bin72 (*S. fumaroxidans* MPOB), AFCMBR_GAC_MaxBin.090 (*M. concilii* GP 6) and AFBR_GAC_MaxBin.001 (*G. lovleyi* SZ) were selected as the references since all the discussed pathways were well reconstructed in these three genomes. The assembled sequences for AFBR GAC were mapped against the three genomes bins to evaluate genes expression levels (Table S4) in AFBR_GAC_Bin72 and AFBR_GAC_MaxBin.001 which were organically recovered from AFBR GAC sample. Meanwhile, the same analysis approach was applied to AFCMBR GAC metatranscriptomic sequences to assess gene expression levels (Table S4) in bin of AFCMBR_GAC_MaxBin.090.

1. **Thermodynamic comparison of the methane production reactions**

To estimate ΔG^0’^of two reactions reducing CO_2_ into methane, the reactions of each step were written and the corresponded ΔG^0’^of each step reactions were calculated (at 298 K, pH 7.0 and all compounds at 1 molar activity) (Table S5) ^2^. Summing up Eq.1 – Eq.7 resulted in an overall reaction of classical CO_2_ reduction, Eq. 8, and ΔG^0’^ of the reaction was obtained by summing up the values from Eq.1 to Eq.7. Considering the differences of electron carriers involved in classical CO_2_ reduction pathway and RHP pathway, the overall reaction of RHP pathway was modified based on the classical CO_2_ reduction reaction by including the involved electron carriers and excluding the uninvolved ones. Specifically, Eq. 9 - Eq. 11 in Table S5 are half reactions of electron carriers, and the reaction of CO_2_ reduction via RHP pathway, Eq. 12 = Eq. 8 + 2 * Eq. 9 – Eq. 10 - Eq. 11, was obtained subsequently.

**3.1 MATLAB programs for thermodynamics calculation**

# Step1: To calculate the contribution from different P_CH4_ and P_CO2_

CH4=[0.00064, 0.00076, 0.00089, 0.00102];

#aqueous concentrations of methane under P_CH4_ at 50%, 60%, 70% and 80%

CO2=[0.01498, 0.01199, 0.00899, 0.00599];

#aqueous concentrations of carbon dioxide under P_CO2_ at 50%, 40%, 30% and 20%

R = 0.008314;

#gas constant

T= 298.15;

#temperature

for i=1:4

k(i)=R x T x ln ((CH4(i))*(CO2(i)));$k\left( i \right)=R \times T \times\ln\left( ({CH}_{4}\left( i \right) \right)\times\left( {CO}_{2}\left( i \right) \right)$

end

#Step 2: To calculate ∆G^0^ for acetoclastic methanogenesis at different acetate concentrations and different P_CH4_ and P_CO2_

acetate=[0.00003,0.0003,0.003, 0.03, 0.3, 3];

#acetate concentrations

∆G^0^_1_=$zeros((length(acetate),length(k));$

for j=1:length(k)

for h=1:length(acetate)

∆G^0^_1_ (h,j)= $\left( -24.84 \right)+k\left( j \right)-R\times T\times ln(acetate(h))$

end

end

#Step 3: To calculate ∆G^0^ for the combined acetoclastic methanogenesis and CO2 reduction pathway at different acetate concentrations and different P_CH4_

∆G^0^_2_=$zeros((length(acetate),length(CH4));$

for j=1:length(CH4)

for h=1:length(acetate)

∆G^0^_2_ (h,j)= $\left( -62.1-24.84 \right)+R \times T \times\ln\left( \left( CH4\left( j \right) \right)\times\left( CH4\left( j \right) \right) \right)- R\times T\times ln(acetate(h))$

#∆G^0^ for the combined acetoclastic methanogenesis and electron-dependent CO2 reduction reaction

end

end

∆G^0^_3_=$zeros((length(acetate),length(CH4));$

for j=1:length(CH4)

for h=1:length(acetate)

∆G^0^_3_ (h,j)= $\left( -29.1-24.84 \right)+R \times T \times\ln\left( \left( CH4\left( j \right) \right)\times\left( CH4\left( j \right) \right) \right)- R\times T\times ln(acetate(h))$

#∆G^0^ for the combined acetoclastic methanogenesis and RHP pathway

end

end

| **Table S1**. Summary of the 4 metagenomes. | | |  | |  | |  | |  | |  |
| --- | --- | --- | --- | --- | --- | --- | --- | --- | --- | --- | --- |
| **Sample** | **Platform** | **Clean Reads** | | **Total sequences (Gbp)** | | **Average length (bp)** | | **# of contigs after assembly** | | **N50 after assembly** | |
| AFBR_GAC | Illumina HiSeq | 157,411,554 | | 53.8 | | 150 | | 543293 | | 1890 | |
| AFBR_GAC hybrid^a^ | Pacbio Sequel | 1,066,949 | | 11.87 | | 5771 | | 495970 | | 2173 | |
| AFCMBR_GAC | Illumina HiSeq | 156,430,090 | | 53.54 | | 150 | | 655739 | | 1707 | |
| AFCMBR_GAC hybrid^a^ | Pacbio Sequel | 507,843 | | 5.34 | | 5700 | | 604865 | | 1885 | |

^a^hybrid sample refers to the assembly of both that trimmed Illumina HiSeq reads and Pacbio Sequel reads.

**Table S2.** High-quality recovered genome bins that are closely related to methanogens, syntrophs and *Geobacter*.

| **Bin name** | **Chromosome size, bp** | **GC content, %** | **Completeness^a^, %** | **Contamination, %** | **Strain heterogeneity, %** | **Phylophlan** |
| --- | --- | --- | --- | --- | --- | --- |
| AFBR_GAC_Bin12 | 3700118 | 48.1 | 100 [69.27] | 0.6 | 0 | *Smithella sp.* F21 |
| AFCMBR_GAC_Bin40 | 3764333 | 48.4 | 100 [97.42] | 29.84 | 16.88 |  |
| AFCMBR_GAC_Bin17 | 3647369 | 55.8 | 92.5 [95.51] | 2.31 | 0 |  |
| AFCMBR_GAC_MaxBin.006 | 3644658 | 48.4 | 100 [96.13] | 13.87 | 3.57 |  |
| AFCMBR_GAC_MaxBin.051 | 3622302 | 58 | 100 [96.13] | 9.44 | 13.64 |  |
| **AFBR_GAC_Bin72** | 4370393 | 59.1 | 100 [81.29] | 7.3 | 13.89 | *Syntrophobacter fumaroxidans* MPOB |
| AFCMBR_GAC_Bin25 | 4738005 | 60.2 | 97.5 [97.26] | 18.04 | 4.17 |  |
| AFBR_GAC_MaxBin.104 | 4482155 | 59.1 | 100% [95.32] | 3.87 | 0 |  |
| AFBR_GAC_MaxBin.008 | 4909591 | 60.1 | 97.5% [99.19] | 10.97 | 3.45 |  |
| AFCMBR_GAC_Bin24 | 3956411 | 52 | 95 [97.71] | 26.47 | 27.59 | *Methanothrix concilii* GP 6 |
| AFBR_GAC_MaxBin.122 | 1446900 | 53.7 | 95% [75.76] | 1.96 | 33.33 |  |
| **AFCMBR_GAC_MaxBin.090** | 4095192 | 51.8 | 97.5% [95.59] | 30.07 | 32.47 |  |
| AFCMBR_GAC_MaxBin.061 | 3035137 | 52.6 | 95% [93.57] | 27.45 | 23.64 |  |
| AFCMBR_GAC_Bin.212 | 5221490 | 55.9 | 95 [83.04] | 37.82 | 34.04 | *Geobacter lovleyi* SZ |
| **AFBR_GAC_MaxBin.001** | 3296093 | 51.4 | 100% [98.35] | 3.39 | 0 |  |
| AFCMBR_GAC_Bin03 | 2080859 | 54.2 | 97.5 [92.37] | 1.33 | 66.67 | *Methanoregula formicica* SMSP DSM |
| AFCMBR_GAC_MaxBin.029 | 2321899 | 53.6 | 100% [97.25] | 0.65 | 0 |  |
| AFCMBR_GAC_Bin21 | 2245848 | 54.8 | 95 [90.5] | 16.97 | 23.81 | *Methanolinea tarda NOBI-1* |
| AFBR_Bulk_Bin.026 | 3639841 | 46.4 | 100 [99.49] | 4.46 | 7.14 | *Syntrophomonas zehnderi* |
| AFCMBR_GAC_MaxBin.071 | 3286932 | 64.2 | 100% [80.17] | 36.14 | 7.22 | *Syntrophus sp GWC2* |

^a^values represents the completeness of genome bins assessed by Maxbin while the values in parenthesis were assessed with CheckM

**Table S3.** Coding DNA sequences (CDS) predicted to be involved in the metabolism of main pathways.

| CDS | Predicted function | Gene name | Gene name | Enzyme commission |
| --- | --- | --- | --- | --- |
| **AFBR_GAC_MaxBin.001- *Geobacter lovleyi* - acetate oxidation to CO_2_ (TCA cycle)** | | | | |
| AFBR_GAC_MaxBin.001_02087 | phosphate acetyltransferase | *pta* | phosphate acetyltransferase | EC:2.3.1.8 |
| AFBR_GAC_MaxBin.001_00587 | acetate kinase | *ackA* | acetate kinase | EC:2.7.2.1 |
| AFBR_GAC_MaxBin.001_00963 | acetyl-CoA C-acetyltransferase | *ato* | acetyl-CoA C-acetyltransferase | EC:2.3.1.9 |
| AFBR_GAC_MaxBin.001_00357 | citrate synthase | *cs* | citrate (Si)-synthase | EC:2.3.3.1 |
| AFBR_GAC_MaxBin.001_01231 | aconitate hydratase 2 / 2-methylisocitrate dehydratase | *acnB* | aconitate hydratase | EC:4.2.1.3 4.2.1.99 |
| AFBR_GAC_MaxBin.001_02542 | aconitate hydratase | *aco* | aconitate hydratase | EC:4.2.1.3 |
| AFBR_GAC_MaxBin.001_01771 | isocitrate dehydrogenase | *idh1* | isocitrate dehydrogenase (NADP+) | EC:1.1.1.42 |
| AFBR_GAC_MaxBin.001_01766 | 2-oxoglutarate ferredoxin oxidoreductase subunit gamma | *korC* | 2-oxoglutarate synthase | EC 1.2.7.3 |
| AFBR_GAC_MaxBin.001_01767 | 2-oxoglutarate/2-oxoacid ferredoxin oxidoreductase subunit beta | *korB* | 2-oxoglutarate synthase | EC:1.2.7.3 1.2.7.11 |
| AFBR_GAC_MaxBin.001_01768 | 2-oxoglutarate/2-oxoacid ferredoxin oxidoreductase subunit alpha | *korA* | 2-oxoglutarate synthase | EC:1.2.7.3 1.2.7.11 |
| AFBR_GAC_MaxBin.001_01769 | 2-oxoglutarate ferredoxin oxidoreductase subunit delta | *korD* | 2-oxoglutarate synthase | EC 1.2.7.3 |
| AFBR_GAC_MaxBin.001_01643 | succinyl-CoA synthetase alpha subunit | *sucD* | succinate---CoA ligase (ADP-forming) | EC 6.2.1.5 |
| AFBR_GAC_MaxBin.001_01644 | succinyl-CoA synthetase beta subunit | *sucC* | succinate---CoA ligase (ADP-forming) | EC 6.2.1.5 |
| AFBR_GAC_MaxBin.001_01720 | succinate dehydrogenase / fumarate reductase, iron-sulfur subunit | sdhB | succinate dehydrogenase | EC 1.3.5.1 |
| AFBR_GAC_MaxBin.001_01721 | succinate dehydrogenase / fumarate reductase, flavoprotein subunit | sdhA | fumarate reductase (quinol) | EC 1.3.5.4 |
| AFBR_GAC_MaxBin.001_01936 | fumarate hydratase | fum | fumarate hydratase | EC:4.2.1.2 |
| AFBR_GAC_MaxBin.001_01770 | malate dehydrogenase | mdh | malate dehydrogenase | EC 1.1.1.37 |
|  |  |  |  |  |
| **AFBR_GAC_Bin72 - *Syntrophobacter fumaroxidans* - methylmalonyl-CoA (MMC) pathway** | | | | |
| AFBR_GAC_MaxBin.072_00988 | propionate CoA transferase | *pct* | propionate CoA-transferase | EC:2.8.3.1 |
| AFBR_GAC_MaxBin.072_03848 | propionyl-CoA:oxaloacetate transcarboxylase | *pot, pccA* | methylmalonyl-CoA carboxytransferase | EC:2.1.3.1 |
| AFBR_GAC_MaxBin.072_00452 | methylmalonyl-CoA/ethylmalonyl-CoA epimerase | *mcee, epi* | methylmalonyl-CoA/ethylmalonyl-CoA epimerase | EC:5.1.99.1 |
| AFBR_GAC_MaxBin.072_00277 | methylmalonyl-CoA mutase | *mcm* | methylmalonyl-CoA mutase | EC:5.4.99.2 |
| AFBR_GAC_MaxBin.072_01546 | succinyl-CoA synthetase | *scs* | succinyl-CoA synthetase alpha subunit | EC:6.2.1.5 |
| AFBR_GAC_MaxBin.072_03945 | succinyl-CoA synthetase | *scs* | succinyl-CoA synthetase alpha subunit | EC:6.2.1.5 |
| AFBR_GAC_MaxBin.072_01935 | succinate dehydrogenase | *sdh/fr* | succinate dehydrogenase | EC:1.3.5.1 1.3.5.4 |
| AFBR_GAC_MaxBin.072_02545 | fumarate hydratase (fumarase) | *fht* | fumarate hydratase | EC:4.2.1.2 |
| AFBR_GAC_MaxBin.072_03984 | malate dehydrogenase | *mdh* | malate dehydrogenase (oxaloacetate-decarboxylating)(NADP+) | EC:1.1.1.40 |
| AFBR_GAC_MaxBin.072_02538 | malate dehydrogenase | *mdh* | malate dehydrogenase (oxaloacetate-decarboxylating)(NADP+) | EC:1.1.1.40 |
| AFBR_GAC_MaxBin.072_02198 | pyruvate dehydrogenase | *pdhD* | pyruvate dehydrogenase E1 component | EC:1.2.4.1 |
| AFBR_GAC_MaxBin.072_02199 | pyruvate dehydrogenase | *pdhB* | pyruvate dehydrogenase E1 component | EC:1.2.4.1 |
| AFBR_GAC_MaxBin.072_02200 | pyruvate dehydrogenase | *dlat* | pyruvate dehydrogenase E2 component (dihydrolipoamide acetyltransferase) | EC:2.3.1.12 |
| AFBR_GAC_MaxBin.072_01790 | 2-oxoglutarate/2-oxoacid ferredoxin oxidoreductase | *korA* | 2-oxoglutarate/2-oxoacid ferredoxin oxidoreductase subunit alpha | EC:1.2.7.3 1.2.7.11 |
| AFBR_GAC_MaxBin.072_00502 | acetyl-CoA synthetas | *acs* | acetyl-CoA synthetase (ADP-forming) | EC:6.2.1.13 |
| AFBR_GAC_MaxBin.072_00503 | carbon-monoxide dehydrogenase | *cooS* | carbon-monoxide dehydrogenase | EC:1.2.7.4 |
|  |  |  |  |  |
| **AFCMBR_GAC_MaxBin.090 - *Methanothrix concilii* - acetoclastic methanogenesis** | | | | |
| AFCMBR_GAC_MaxBin.090_00490 | acetyl-CoA synthetase | *acs* | acetyl-CoA synthetase | EC:6.2.1.1 |
| AFCMBR_GAC_MaxBin.090_00611 | acetyl-CoA synthetase | *Acs* | acetyl-CoA synthetase | EC:6.2.1.1 |
| AFCMBR_GAC_MaxBin.090_00613 | acetyl-CoA synthetase | *acs* | acetyl-CoA synthetase | EC:6.2.1.1 |
| AFCMBR_GAC_MaxBin.090_01997 | acetyl-CoA synthetase | *acs* | acetyl-CoA synthetase | EC:6.2.1.1 |
| AFCMBR_GAC_MaxBin.090_01578 | acetyl-CoA decarbonylase/synthase | *cdhA* | acetyl-CoA decarbonylase/synthase | EC:1.2.7.4 |
| AFCMBR_GAC_MaxBin.090_01577 | acetyl-CoA decarbonylase/synthase | *cdhB* | acetyl-CoA decarbonylase/synthase | EC:1.2.7.4 |
| AFCMBR_GAC_MaxBin.090_01576 | acetyl-CoA decarbonylase/synthase | *cdhC* | acetyl-CoA decarbonylase/synthase | EC:2.3.1.- |
| AFCMBR_GAC_MaxBin.090_01573 | acetyl-CoA decarbonylase/synthase | *cdhD* | acetyl-CoA decarbonylase/synthase | EC:2.1.1.245 |
| AFCMBR_GAC_MaxBin.090_01572 | acetyl-CoA decarbonylase/synthase | *cdhE* | acetyl-CoA decarbonylase/synthase | EC:2.1.1.24 |
| AFCMBR_GAC_MaxBin.090_00058 | methyl-H4MPT coenzyme M methyltransferase | *mtrA* | tetrahydromethanopterin S-methyltransferase | EC:2.1.1.86 |
| AFCMBR_GAC_MaxBin.090_00057 | methyl-H4MPT coenzyme M methyltransferase | *mtrB* | tetrahydromethanopterin S-methyltransferase | EC:2.1.1.86 |
| AFCMBR_GAC_MaxBin.090_00056 | methyl-H4MPT coenzyme M methyltransferase | *mtrC* | tetrahydromethanopterin S-methyltransferase | EC:2.1.1.86 |
| AFCMBR_GAC_MaxBin.090_00055 | methyl-H4MPT coenzyme M methyltransferase | *mtrD* | tetrahydromethanopterin S-methyltransferase | EC:2.1.1.86 |
| AFCMBR_GAC_MaxBin.090_00054 | methyl-H4MPT coenzyme M methyltransferase | *mtrE* | tetrahydromethanopterin S-methyltransferase | EC:2.1.1.86 |
| AFCMBR_GAC_MaxBin.090_00059 | methyl-H4MPT coenzyme M methyltransferase | *mtrF* | tetrahydromethanopterin S-methyltransferase | EC:2.1.1.86 |
| AFCMBR_GAC_MaxBin.090_00060 | methyl-H4MPT coenzyme M methyltransferase | *mtrG* | tetrahydromethanopterin S-methyltransferase | EC:2.1.1.86 |
| AFCMBR_GAC_MaxBin.090_00061 | methyl-H4MPT coenzyme M methyltransferase | *mtrH* | tetrahydromethanopterin S-methyltransferase | EC:2.1.1.86 |
| AFCMBR_GAC_MaxBin.090_00918 | methyl-CoM methylreductase | *mcr* | methyl-CoM reductase | EC:2.8.4.1 |
| AFCMBR_GAC_MaxBin.090_02073 | heterodisulfide reductase subunit A | *hdrA* | dihydromethanophenazine:CoB-CoM heterodisulfide reductase | EC:1.8.98.1 |
| AFCMBR_GAC_MaxBin.090_02077 | heterodisulfide reductase subunit B | *hdrB* | dihydromethanophenazine:CoB-CoM heterodisulfide reductase | EC:1.8.98.1 |
| AFCMBR_GAC_MaxBin.090_02076 | heterodisulfide reductase subunit C | *hdrC* | dihydromethanophenazine:CoB-CoM heterodisulfide reductase | EC:1.8.98.1 |
| AFCMBR_GAC_MaxBin.090_01697 | heterodisulfide reductase subunit D | *hdrD* | dihydromethanophenazine:CoB-CoM heterodisulfide reductase | EC:1.8.98.1 |
| AFCMBR_GAC_MaxBin.090_01698 | heterodisulfide reductase subunit E | *hdrE* | dihydromethanophenazine:CoB-CoM heterodisulfide reductase | EC:1.8.98.1 |
| AFCMBR_GAC_MaxBin.090_00779 | inorganic pyrophosphatase | *ppase* | inorganic diphosphatase | EC 3.6.1.1 |
| AFCMBR_GAC_MaxBin.090_03846 | inorganic pyrophosphatase | *ppase* | inorganic diphosphatase | EC 3.6.1.1 |
| AFCMBR_GAC_MaxBin.090_00723 | carbonic anhydrase | *ca* | carbonic anhydrase | EC 4.2.1.1 |
| AFCMBR_GAC_MaxBin.090_03493 | carbonic anhydrase | *ca* | carbonic anhydrase | EC 4.2.1.1 |
| AFCMBR_GAC_MaxBin.090_03897 | carbonic anhydrase | *ca* | carbonic anhydrase | EC 4.2.1.1 |
| **substrate transporter & energy related** | | | | |
| AFCMBR_GAC_MaxBin.090_00065 | TC.SSS/putative acetate transporter | *ady2* | putative acetate transporter | / |
| AFCMBR_GAC_MaxBin.090_02156 | TC.SSS/putative acetate transporter | *ady2* | putative acetate transporter | / |
| AFCMBR_GAC_MaxBin.090_02179 | TC.SSS/putative acetate transporter | *ady2* | putative acetate transporter | / |
| AFCMBR_GAC_MaxBin.090_01416 | energy-conserving hydrogenases | *ehb* | energy-conserving hydrogenases | / |
| AFCMBR_GAC_MaxBin.090_04135 | energy-conserving hydrogenases | *ehb* | energy-conserving hydrogenases | / |
| AFCMBR_GAC_MaxBin.090_00638 | F-type H+-transporting ATPase subunit a | *ATPS* | H+-transporting ATPase | EC:3.6.3.14 |
| AFCMBR_GAC_MaxBin.090_02459 | F-type H+-transporting ATPase subunit a | *ATPS* | H+-transporting ATPase | EC:3.6.3.14 |
| AFCMBR_GAC_MaxBin.090_02456 | F-type H+-transporting ATPase subunit alpha | *ATPS* | H+-transporting ATPase | EC:3.6.3.14 |
| AFCMBR_GAC_MaxBin.090_00641 | F-type H+-transporting ATPase subunit alpha | *ATPS* | H+-transporting ATPase | EC:3.6.3.14 |
| AFCMBR_GAC_MaxBin.090_02457 | F-type H+-transporting ATPase subunit b | *ATPS* | H+-transporting ATPase | EC:3.6.3.14 |
| AFCMBR_GAC_MaxBin.090_00640 | F-type H+-transporting ATPase subunit b | *ATPS* | H+-transporting ATPase | EC:3.6.3.14 |
| AFCMBR_GAC_MaxBin.090_00634 | F-type H+-transporting ATPase subunit beta | *ATPS* | H+-transporting ATPase | EC:3.6.3.14 |
| AFCMBR_GAC_MaxBin.090_02463 | F-type H+-transporting ATPase subunit beta | *ATPS* | H+-transporting ATPase | EC:3.6.3.14 |
| AFCMBR_GAC_MaxBin.090_00639 | F-type H+-transporting ATPase subunit c | *ATPS* | H+-transporting ATPase | EC:3.6.3.14 |
| AFCMBR_GAC_MaxBin.090_02458 | F-type H+-transporting ATPase subunit c | *ATPS* | H+-transporting ATPase | EC:3.6.3.14 |
| AFCMBR_GAC_MaxBin.090_02462 | F-type H+-transporting ATPase subunit epsilon | *ATPS* | H+-transporting ATPase | EC:3.6.3.14 |
| AFCMBR_GAC_MaxBin.090_00635 | F-type H+-transporting ATPase subunit epsilon | *ATPS* | H+-transporting ATPase | EC:3.6.3.14 |
| AFCMBR_GAC_MaxBin.090_00642 | F-type H+-transporting ATPase subunit gamma | *ATPS* | H+-transporting ATPase | EC:3.6.3.14 |
| AFCMBR_GAC_MaxBin.090_02455 | F-type H+-transporting ATPase subunit gamma | *ATPS* | H+-transporting ATPase | EC:3.6.3.14 |
|  |  |  |  |  |
| **AFCMBR_GAC_MaxBin.090 - *Methanothirx concilii* - RHP pathway** | | | | |
| AFCMBR_GAC_MaxBin.090_00783 | phosphoribulokinase | *prk* | phosphoribulokinase | EC:2.7.1.19 |
| AFCMBR_GAC_MaxBin.090_02729 | ribulose-bisphosphate carboxylase large chain | *ruBisCO* | ribulose-bisphosphate carboxylase | EC 4.1.1.39 |
| AFCMBR_GAC_MaxBin.090_00965 | 3-phosphoglycerate kinase | *pgk* | phosphoglycerate kinase | EC:2.7.2.3 |
| AFCMBR_GAC_MaxBin.090_02093 | glyceraldehyde-3-phosphate dehydrogenase (NAD(P)+) (phosphorylating) | *gap2* | glyceraldehyde-3-phosphate dehydrogenase (NAD(P)+) (phosphorylating); | EC:1.2.1.59 |
| AFCMBR_GAC_MaxBin.090_02711 | glyceraldehyde-3-phosphate dehydrogenase (NAD(P)+) (phosphorylating) | *gap2* | glyceraldehyde-3-phosphate dehydrogenase (NAD(P)+) (phosphorylating); | EC:1.2.1.59 |
| AFCMBR_GAC_MaxBin.090_00498 | fructose-bisphosphate aldolase | *aldolase* | fructose-bisphosphate aldolase | EC 4.1.2.13 |
| AFCMBR_GAC_MaxBin.090_00256 | fructose-1,6- bisphosphatase | *fbpase* | fructose-bisphosphatase | EC 3.1.3.11 |
| AFCMBR_GAC_MaxBin.090_04006 | fructose-1,6- bisphosphatase | *fbpase* | fructose-bisphosphatase | EC 3.1.3.11 |
| AFCMBR_GAC_MaxBin.090_01312 | 6-phospho-3-hexuloisomerase | *phi* | 6-phospho-3-hexuloisomerase | EC 5.3.1.27 |
| AFCMBR_GAC_MaxBin.090_01348 | 3-hexulose-6-phosphate synthase | *hps* | 3-hexulose-6-phosphate synthase | EC:4.2.1.147 4.1.2.43 |
| AFCMBR_GAC_MaxBin.090_02991 | 3-hexulose-6-phosphate synthase | *hps* | 3-hexulose-6-phosphate synthase | EC:4.2.1.147 4.1.2.43 |
| AFCMBR_GAC_MaxBin.090_04145 | 3-hexulose-6-phosphate synthase | *hps* | 3-hexulose-6-phosphate synthase | EC:4.2.1.147 4.1.2.43 |
| AFCMBR_GAC_MaxBin.090_00600 | AMP phosphorylase | *AMPpase* | AMP phosphorylase | EC:2.4.2.57 |
| AFCMBR_GAC_MaxBin.090_01365 | ribose-5-phosphate isomerase | *riBP isomerase* | ribose-5-phosphate isomerase | EC 5.3.1.6 |
| AFCMBR_GAC_MaxBin.090_01068 | 5,6,7,8-tetrahydromethanopterin hydro-lyase | *fae* | 5,6,7,8-tetrahydromethanopterin hydro-lyase | EC:4.2.1.147 |
| AFCMBR_GAC_MaxBin.090_01348 | 5,6,7,8-tetrahydromethanopterin hydro-lyase | *fae* | 5,6,7,8-tetrahydromethanopterin hydro-lyase | EC:4.2.1.147 |
| AFCMBR_GAC_MaxBin.090_02991 | 5,6,7,8-tetrahydromethanopterin hydro-lyase | *fae* | 5,6,7,8-tetrahydromethanopterin hydro-lyase | EC:4.2.1.147 |
| AFCMBR_GAC_MaxBin.090_04145 | 5,6,7,8-tetrahydromethanopterin hydro-lyase | *fae* | 5,6,7,8-tetrahydromethanopterin hydro-lyase | EC:4.2.1.147 |
|  |  |  |  |  |
| **AFCMBR_GAC_MaxBin.090-*Methanothrix concilii-*CO2 reduction** | | | | |
| AFCMBR_GAC_MaxBin.090_01093 | formylmethanofuran dehydrogenase | *fwd* | formylmethanofuran dehydrogenase | EC:1.2.7.12 |
| AFCMBR_GAC_MaxBin.090_01090 | formylmethanofuran dehydrogenase | *fwd* | formylmethanofuran dehydrogenase | EC:1.2.7.12 |
| AFCMBR_GAC_MaxBin.090_00501 | formylmethanofuran dehydrogenase | *fwd* | formylmethanofuran dehydrogenase | EC:1.2.7.12 |
| AFCMBR_GAC_MaxBin.090_01091 | formylmethanofuran dehydrogenase | *fwd* | formylmethanofuran dehydrogenase | EC:1.2.7.12 |
| AFCMBR_GAC_MaxBin.090_01258 | formylmethanofuran dehydrogenase | *fwd* | formylmethanofuran dehydrogenase | EC:1.2.7.12 |
| AFCMBR_GAC_MaxBin.090_01278 | formyl methanofuran–H4MPT formyltransferase | *ftr* | formylmethanofuran---tetrahydromethanopterin N-formyltransferase; | EC:2.3.1.101 |
| AFCMBR_GAC_MaxBin.090_00012 | methenyl-H4MPT cyclohydrolase | *mch* | methenyltetrahydromethanopterin cyclohydrolase | EC:3.5.4.27 |
| AFCMBR_GAC_MaxBin.090_00079 | F420-dependent methylene-H4MPT dehydrogenase | *mtd* | methylenetetrahydromethanopterin dehydrogenase | EC:1.5.98.1 |
| AFCMBR_GAC_024_02542 | F420-dependent methylene H4MPT reductase | *mer* | 5,10-methylenetetrahydromethanopterin reductase | EC:1.5.98.2 |
| AFCMBR_GAC_MaxBin.090_03089 | coenzyme F420 hydrogenase | *frh* | coenzyme F420 hydrogenase | EC:1.12.98.1 |
| AFCMBR_GAC_MaxBin.090_03298 | coenzyme F420 hydrogenase | *frh* | coenzyme F420 hydrogenase | EC:1.12.98.1 |
| AFCMBR_GAC_MaxBin.090_00058 | tetrahydromethanopterin S-methyltransferase subunit A | *mtrA* | tetrahydromethanopterin S-methyltransferase | EC:2.1.1.86 |
| AFCMBR_GAC_MaxBin.090_00057 | tetrahydromethanopterin S-methyltransferase subunit B | *mtrB* | tetrahydromethanopterin S-methyltransferase | EC:2.1.1.86 |
| AFCMBR_GAC_MaxBin.090_00056 | tetrahydromethanopterin S-methyltransferase subunit C | *mtrC* | tetrahydromethanopterin S-methyltransferase | EC:2.1.1.86 |
| AFCMBR_GAC_MaxBin.090_00055 | tetrahydromethanopterin S-methyltransferase subunit D | *mtrD* | tetrahydromethanopterin S-methyltransferase | EC:2.1.1.86 |
| AFCMBR_GAC_MaxBin.090_00054 | tetrahydromethanopterin S-methyltransferase subunit E | *mtrE* | tetrahydromethanopterin S-methyltransferase | EC:2.1.1.86 |
| AFCMBR_GAC_MaxBin.090_00059 | tetrahydromethanopterin S-methyltransferase subunit F | *mtrF* | tetrahydromethanopterin S-methyltransferase | EC:2.1.1.86 |
| AFCMBR_GAC_MaxBin.090_00060 | tetrahydromethanopterin S-methyltransferase subunit G | *mtrG* | tetrahydromethanopterin S-methyltransferase | EC:2.1.1.86 |
| AFCMBR_GAC_MaxBin.090_00061 | tetrahydromethanopterin S-methyltransferase subunit H | *mtrH* | tetrahydromethanopterin S-methyltransferase | EC:2.1.1.86 |
| AFCMBR_GAC_MaxBin.090_00987 | methyl-coenzyme M reductase alpha subunit | *mcrA* | coenzyme-B sulfoethylthiotransferase | EC:2.8.4.1 |
| AFCMBR_GAC_MaxBin.090_00984 | methyl-coenzyme M reductase beta subunit | *mcrB* | coenzyme-B sulfoethylthiotransferase | EC:2.8.4.1 |
| AFCMBR_GAC_MaxBin.090_00918 | methyl-coenzyme M reductase subunit C | *mcrC* | coenzyme-B sulfoethylthiotransferase | EC:2.8.4.1 |
| AFCMBR_GAC_MaxBin.090_00985 | methyl-coenzyme M reductase subunit D | *mcrD* | coenzyme-B sulfoethylthiotransferase | EC:2.8.4.1 |
| AFCMBR_GAC_MaxBin.090_00986 | methyl-coenzyme M reductase gamma subunit | *mcrG* | coenzyme-B sulfoethylthiotransferase | EC:2.8.4.1 |
| AFCMBR_GAC_MaxBin.090_03695 | methyl-coenzyme M reductase gamma subunit | *mcrG* | coenzyme-B sulfoethylthiotransferase | EC:2.8.4.1 |
| AFCMBR_GAC_MaxBin.090_02073 | heterodisulfide reductase subunit A | *hdrA* | dihydromethanophenazine:CoB-CoM heterodisulfide reductase | EC:1.8.98.1 |
| AFCMBR_GAC_MaxBin.090_02077 | heterodisulfide reductase subunit B | *hdrB* | dihydromethanophenazine:CoB-CoM heterodisulfide reductase | EC:1.8.98.1 |
| AFCMBR_GAC_MaxBin.090_02076 | heterodisulfide reductase subunit C | *hdrC* | dihydromethanophenazine:CoB-CoM heterodisulfide reductase | EC:1.8.98.1 |
| AFCMBR_GAC_MaxBin.090_01697 | heterodisulfide reductase subunit D | *hdrD* | dihydromethanophenazine:CoB-CoM heterodisulfide reductase | EC:1.8.98.1 |
| AFCMBR_GAC_MaxBin.090_01698 | heterodisulfide reductase subunit E | *hdrE* | dihydromethanophenazine:CoB-CoM heterodisulfide reductase | EC:1.8.98.1 |

**Table S4.** Genes expression of the main pathways (propionate degradation, methane generation) in *S.fumaroxidans* MPOB, *M. concilii* GP 6 and *G. lovleyi* SZ.

| **Function** | **Gene Abbrev.** | **CDS** | **log2RPKM** |
| --- | --- | --- | --- |
| **Acetate oxidation to CO_2_ (TCA cycle)** | | | |
| phosphate acetyltransferase | *pta* | AFBR_GAC_MaxBin.001_02087 | 5.47 |
| acetate kinase | *ackA* | AFBR_GAC_MaxBin.001_00587 | 7.45 |
| acetyl-CoA C-acetyltransferase | *ato* | AFBR_GAC_MaxBin.001_00963 | 4.79 |
| citrate synthase | *cs* | AFBR_GAC_MaxBin.001_00357 | 5.06 |
| aconitate hydratase | *aco* | AFBR_GAC_MaxBin.001_01231 | 5.13 |
| aconitate hydratase | *aco* | AFBR_GAC_MaxBin.001_02542 | 4.51 |
| isocitrate dehydrogenase | *idh1* | AFBR_GAC_MaxBin.001_01771 | 5.31 |
| 2-oxoglutarate/2-oxoacid ferredoxin oxidoreductase | *kor* | AFBR_GAC_MaxBin.001_02544 | 4.61 |
| succinyl-CoA synthetase alpha subunit | *suc* | AFBR_GAC_MaxBin.001_01643 | 5.66 |
| succinyl-CoA synthetase beta subunit | *suc* | AFBR_GAC_MaxBin.001_01644 | 5.25 |
| succinate dehydrogenase / fumarate reductase | *frd* | AFBR_GAC_MaxBin.001_01721 | 4.53 |
| fumarate hydratase | *fum* | AFBR_GAC_MaxBin.001_01936 | 4.77 |
| malate dehydrogenase | *mdh* | AFBR_GAC_MaxBin.001_01770 | 8.30 |
|  |  |  |  |
| **Methylmalonyl-CoA (MMC) pathway** | | | |
| propionate CoA transferase | *pct* | AFBR_GAC_MaxBin.072_00988 | 7.11 |
| propionyl-CoA:oxaloacetate transcarboxylase | *pot, pccA* | AFBR_GAC_MaxBin.072_03848 | 6.50 |
| methylmalonyl-CoA/ethylmalonyl-CoA epimerase | *mcee, epi* | AFBR_GAC_MaxBin.072_00452 | 8.40 |
| methylmalonyl-CoA mutase | *mcm* | AFBR_GAC_MaxBin.072_00277 | 8.24 |
| succinyl-CoA synthetase | *scs* | AFBR_GAC_MaxBin.072_01546 | 10.13 |
| succinyl-CoA synthetase | *scs* | AFBR_GAC_MaxBin.072_03945 | 7.32 |
| succinate dehydrogenase | *sdh/fr (2)* | AFBR_GAC_MaxBin.072_01935 | 6.39 |
| fumarate hydratase (fumarase) | *fht* | AFBR_GAC_MaxBin.072_02545 | 8.76 |
| malate dehydrogenase | *mdh* | AFBR_GAC_MaxBin.072_03984 | 8.37 |
| malate dehydrogenase | *mdh* | AFBR_GAC_MaxBin.072_02538 | 5.95 |
| pyruvate dehydrogenase | *pdh* | AFBR_GAC_MaxBin.072_02198 | 5.41 |
| pyruvate dehydrogenase | *pdh* | AFBR_GAC_MaxBin.072_02200 | 5.17 |
| 2-oxoglutarate/2-oxoacid ferredoxin oxidoreductase | *korA* | AFBR_GAC_MaxBin.072_01790 | 5.37 |
| acetyl-CoA synthetas | *acs* | AFBR_GAC_MaxBin.072_00502 | 8.72 |
| carbon-monoxide dehydrogenase | *cooS* | AFBR_GAC_MaxBin.072_00503 | 6.89 |
|  |  |  |  |
| **Acetoclastic methanogenesis** |  |  |  |
| acetyl-CoA synthetase | *acs* | AFCMBR_GAC_MaxBin.090_00490 | 7.57 |
| acetyl-CoA synthetase | *acs* | AFCMBR_GAC_MaxBin.090_00611 | 11.96 |
| acetyl-CoA synthetase | *acs* | AFCMBR_GAC_MaxBin.090_00613 | 11.26 |
| acetyl-CoA synthetase | *acs* | AFCMBR_GAC_MaxBin.090_01997 | 6.51 |
| acetyl-CoA synthetase | *acs* | AFCMBR_GAC_MaxBin.090_03370 | 10.16 |
| acetyl-CoA decarbonylase/synthase | *cdh* | AFCMBR_GAC_MaxBin.090_01578 | 11.13 |
| acetyl-CoA decarbonylase/synthase | *cdh* | AFCMBR_GAC_MaxBin.090_01577 | 11.13 |
| acetyl-CoA decarbonylase/synthase | *cdh* | AFCMBR_GAC_MaxBin.090_01576 | 11.48 |
| acetyl-CoA decarbonylase/synthase | *cdh* | AFCMBR_GAC_MaxBin.090_01573 | 11.06 |
| acetyl-CoA decarbonylase/synthase | *cdh* | AFCMBR_GAC_MaxBin.090_01572 | 11.41 |
| Carbon monoxide dehydrogenase | *cdh* | AFCMBR_GAC_MaxBin.090_00633 | 8.61 |
| Carbon monoxide dehydrogenase | *cdh* | AFCMBR_GAC_MaxBin.090_03405 | 8.82 |
| methyl-H4MPT coenzyme M methyltransferase | *mtr* | AFCMBR_GAC_MaxBin.090_00058 | 10.27 |
| methyl-H4MPT coenzyme M methyltransferase | *mtr* | AFCMBR_GAC_MaxBin.090_00057 | 10.22 |
| methyl-H4MPT coenzyme M methyltransferase | *mtr* | AFCMBR_GAC_MaxBin.090_00056 | 10.22 |
| methyl-H4MPT coenzyme M methyltransferase | *mtr* | AFCMBR_GAC_MaxBin.090_00055 | 10.15 |
| methyl-H4MPT coenzyme M methyltransferase | *mtr* | AFCMBR_GAC_MaxBin.090_00054 | 10.96 |
| methyl-H4MPT coenzyme M methyltransferase | *mtr* | AFCMBR_GAC_MaxBin.090_00059 | 9.04 |
| methyl-H4MPT coenzyme M methyltransferase | *mtr* | AFCMBR_GAC_MaxBin.090_00060 | 10.74 |
| methyl-H4MPT coenzyme M methyltransferase | *mtr* | AFCMBR_GAC_MaxBin.090_00061 | 9.91 |
| methyl-CoM methylreductase | *mcr* | AFCMBR_GAC_MaxBin.090_00918 | 9.70 |
| heterodisulfide reductase subunit A | *hdr* | AFCMBR_GAC_MaxBin.090_02073 | 9.14 |
| heterodisulfide reductase subunit B | *hdr* | AFCMBR_GAC_MaxBin.090_02077 | 6.16 |
| heterodisulfide reductase subunit C | *hdr* | AFCMBR_GAC_MaxBin.090_02076 | 7.83 |
| heterodisulfide reductase subunit D | *hdr* | AFCMBR_GAC_MaxBin.090_01697 | 7.04 |
| heterodisulfide reductase subunit E | *hdr* | AFCMBR_GAC_MaxBin.090_01698 | 9.90 |
| inorganic pyrophosphatase | *ppase* | AFCMBR_GAC_MaxBin.090_00779 | 9.41 |
| inorganic pyrophosphatase | *ppase* | AFCMBR_GAC_MaxBin.090_03846 | 11.29 |
| carbonic anhydrase | *ca* | AFCMBR_GAC_MaxBin.090_03897 | 6.54 |
| **substrate transporter & energy related** |  |  |  |
| TC.SSS/putative acetate transporter |  | AFCMBR_GAC_MaxBin.090_02156 | 6.24 |
| TC.SSS/putative acetate transporter |  | AFCMBR_GAC_MaxBin.090_02179 | 7.80 |
| F-type H+-transporting ATPase subunit alpha |  | AFCMBR_GAC_MaxBin.090_02456 | 7.31 |
| F-type H+-transporting ATPase subunit b |  | AFCMBR_GAC_MaxBin.090_02457 | 6.26 |
| F-type H+-transporting ATPase subunit beta |  | AFCMBR_GAC_MaxBin.090_00634 | 5.45 |
| F-type H+-transporting ATPase subunit beta |  | AFCMBR_GAC_MaxBin.090_02463 | 5.45 |
| F-type H+-transporting ATPase subunit gamma |  | AFCMBR_GAC_MaxBin.090_02455 | 7.09 |
| F-type H+-transporting ATPase subunit c |  | AFCMBR_GAC_MaxBin.090_02458 | 7.81 |
| energy-conserving hydrogenases |  | AFCMBR_GAC_MaxBin.090_01416 | 6.50 |
| energy-conserving hydrogenases |  | AFCMBR_GAC_MaxBin.090_04135 | 8.97 |
|  |  |  |  |
| **The RHP pathway** |  |  |  |
| phosphoribulokinase | *prk* | AFCMBR_GAC_MaxBin.090_00783 | 6.95 |
| ribulose-bisphosphate carboxylase large chain | *ruBisCO* | AFCMBR_GAC_MaxBin.090_02729 | 6.05 |
| ribulose-bisphosphate carboxylase large chain | *ruBisCO* | AFCMBR_GAC_MaxBin.090_02062 | 7.72 |
| 3-phosphoglycerate kinase | *pgk* | AFCMBR_GAC_MaxBin.090_00965 | 6.62 |
| glyceraldehyde-3-phosphate dehydrogenase (NAD(P)+) (phosphorylating) | *gapdh* | AFCMBR_GAC_MaxBin.090_02093 | 6.70 |
| glyceraldehyde-3-phosphate dehydrogenase (NAD(P)+) (phosphorylating) | *gapdh* | AFCMBR_GAC_MaxBin.090_02711 | 9.79 |
| fructose-bisphosphate aldolase | *aldo* | AFCMBR_GAC_MaxBin.090_00498 | 6.99 |
| fructose-bisphosphate aldolase | *aldo* | AFCMBR_GAC_MaxBin.090_00257 | 7.75 |
| fructose-1,6- bisphosphatase | *fbp* | AFCMBR_GAC_MaxBin.090_00256 | 8.41 |
| fructose-1,6- bisphosphatase | *Fbp* | AFCMBR_GAC_MaxBin.090_04006 | 9.52 |
| fructose-bisphosphate aldolase | *Fbp* | AFCMBR_GAC_MaxBin.090_01228 | 7.19 |
| 6-phospho-3-hexuloisomerase | *phi* | AFCMBR_GAC_MaxBin.090_01312 | 7.89 |
| 3-hexulose-6-phosphate synthase | *hps* | AFCMBR_GAC_MaxBin.090_01348 | 8.66 |
| 3-hexulose-6-phosphate synthase | *hps* | AFCMBR_GAC_MaxBin.090_02991 | 7.54 |
| 3-hexulose-6-phosphate synthase | *hps* | AFCMBR_GAC_MaxBin.090_04145 | 7.75 |
| 3-hexulose-6-phosphate synthase | *hps* | AFCMBR_GAC_MaxBin.090_00019 | 7.24 |
| AMP phosphorylase | *amp* | AFCMBR_GAC_MaxBin.090_00600 | 9.40 |
| ribose-5-phosphate isomerase | *ribp* | AFCMBR_GAC_MaxBin.090_01365 | 6.44 |
| 5,6,7,8-tetrahydromethanopterin hydro-lyase | *fae* | AFCMBR_GAC_MaxBin.090_01348 | 8.66 |
| 5,6,7,8-tetrahydromethanopterin hydro-lyase | *fae* | AFCMBR_GAC_MaxBin.090_02991 | 7.54 |
| 5,6,7,8-tetrahydromethanopterin hydro-lyase | *fae* | AFCMBR_GAC_MaxBin.090_04145 | 7.75 |
|  |  |  |  |
| **Classical CO_2_ reduction** |  |  |  |
| formylmethanofuran dehydrogenase | *fwd* | AFCMBR_GAC_MaxBin.090_01093 | 8.97 |
| formylmethanofuran dehydrogenase | *fwd* | AFCMBR_GAC_MaxBin.090_01090 | 8.52 |
| formylmethanofuran dehydrogenase | *fwd* | AFCMBR_GAC_MaxBin.090_01091 | 9.68 |
| formylmethanofuran dehydrogenase | *fwd* | AFCMBR_GAC_MaxBin.090_00242 | 5.89 |
| formylmethanofuran dehydrogenase | *fwd* | AFCMBR_GAC_MaxBin.090_00315 | 6.47 |
| formylmethanofuran dehydrogenase | *fwd* | AFCMBR_GAC_MaxBin.090_01781 | 5.84 |
| formylmethanofuran dehydrogenase | *fwd* | AFCMBR_GAC_MaxBin.090_01923 | 6.52 |
| formylmethanofuran dehydrogenase | *fwd* | AFCMBR_GAC_MaxBin.090_02023 | 7.61 |
| formylmethanofuran dehydrogenase | *fwd* | AFCMBR_GAC_MaxBin.090_00007 | 9.03 |
| formyl methanofuran–H4MPT formyltransferase | *ftr* | AFCMBR_GAC_MaxBin.090_01278 | 6.08 |
| formyl methanofuran–H4MPT formyltransferase | *ftr* | AFCMBR_GAC_MaxBin.090_01924 | 7.14 |
| formyl methanofuran–H4MPT formyltransferase | *ftr* | AFCMBR_GAC_MaxBin.090_01925 | 6.28 |
| formyl methanofuran–H4MPT formyltransferase | *ftr* | AFCMBR_GAC_MaxBin.090_01092 | 6.24 |
| formyl methanofuran–H4MPT formyltransferase | *ftr* | AFCMBR_GAC_MaxBin.090_01093 | 8.97 |
| formyl methanofuran–H4MPT formyltransferase | *ftr* | AFCMBR_GAC_MaxBin.090_01278 | 6.08 |
| methenyl-H4MPT cyclohydrolase | *mch* | AFCMBR_GAC_MaxBin.090_00012 | 9.17 |
| F420-dependent methylene-H4MPT dehydrogenase | *mtd* | AFCMBR_GAC_MaxBin.090_00079 | 7.77 |
| 5,10-methylenetetrahydromethanopterin reductase, mer | *mer* | AFCMBR_GAC_024_02542 | 7.96 |
| coenzyme F420 hydrogenase | *frh* | AFCMBR_GAC_MaxBin.090_03089 | 9.78 |
| tetrahydromethanopterin S-methyltransferase subunit A | *mtr* | AFCMBR_GAC_MaxBin.090_00058 | 10.27 |
| tetrahydromethanopterin S-methyltransferase subunit B | *mtr* | AFCMBR_GAC_MaxBin.090_00057 | 10.22 |
| tetrahydromethanopterin S-methyltransferase subunit C | *mtr* | AFCMBR_GAC_MaxBin.090_00056 | 10.22 |
| tetrahydromethanopterin S-methyltransferase subunit D | *mtr* | AFCMBR_GAC_MaxBin.090_00055 | 10.15 |
| tetrahydromethanopterin S-methyltransferase subunit E | *mtr* | AFCMBR_GAC_MaxBin.090_00054 | 10.96 |
| tetrahydromethanopterin S-methyltransferase subunit F | *mtr* | AFCMBR_GAC_MaxBin.090_00059 | 9.04 |
| tetrahydromethanopterin S-methyltransferase subunit G | *mtr* | AFCMBR_GAC_MaxBin.090_00060 | 10.74 |
| tetrahydromethanopterin S-methyltransferase subunit H | *mtr* | AFCMBR_GAC_MaxBin.090_00061 | 9.91 |
| methyl-coenzyme M reductase alpha subunit | *mcr* | AFCMBR_GAC_MaxBin.090_00987 | 12.08 |
| methyl-coenzyme M reductase beta subunit | *mcr* | AFCMBR_GAC_MaxBin.090_00984 | 12.03 |
| methyl-coenzyme M reductase subunit C | *mcr* | AFCMBR_GAC_MaxBin.090_00918 | 9.70 |
| methyl-coenzyme M reductase subunit D | *mcr* | AFCMBR_GAC_MaxBin.090_00985 | 8.90 |
| methyl-coenzyme M reductase gamma subunit | *mcr* | AFCMBR_GAC_MaxBin.090_00986 | 10.75 |
| methyl-coenzyme M reductase gamma subunit | *mcr* | AFCMBR_GAC_MaxBin.090_03695 | 11.68 |
| heterodisulfide reductase subunit A | *hdr* | AFCMBR_GAC_MaxBin.090_02073 | 9.14 |
| heterodisulfide reductase subunit B | *hdr* | AFCMBR_GAC_MaxBin.090_02077 | 6.16 |
| heterodisulfide reductase subunit C | *hdr* | AFCMBR_GAC_MaxBin.090_02076 | 7.83 |
| heterodisulfide reductase subunit D | *hdr* | AFCMBR_GAC_MaxBin.090_01697 | 7.04 |
| heterodisulfide reductase subunit E | *hdr* | AFCMBR_GAC_MaxBin.090_01698 | 9.90 |

**Table S5.** The step reactions involved in classical CO_2_ reduction, half reactions of electron carriers and their standard Gibbs free energy changes (∆G^0'^) at 298 K, pH 7.0 and all compounds at 1 molar activity.

| **Reaction number** | **Reaction** | **∆G^0^' (kJ/mol)** |
| --- | --- | --- |
| Eq. 1 | CO_2_ (aq) + MFR + 2 Fd_red_^2–^ + 2 H^+^ → CHO-MFR + 2 Fd_ox_ + H_2_O | -8.16 |
| Eq. 2 | CHO-MFR + H_4_MPT → CHO‑H_4_MPT + MFR | –5 |
| Eq. 3 | CHO‑H_4_MPT + H^+^ → CH≡H_4_MPT^+^ + H_2_O | –5 |
| Eq. 4 | CH≡H_4_MPT^+^ + F_420_H_2_ → CH_2_=H_4_MPT + F_420_ + H^+^ | 6 |
| Eq. 5 | CH2=H_4_MPT + F_420_H_2_ → CH_3_-H_4_MPT + F_420_ | -6 |
| Eq. 6 | CH_3_‑H_4_MPT + HS‑CoM → CH_3_S-CoM + H_4_MPT | –30 |
| Eq. 7 | CH_3_‑S-CoM + HS‑CoB → CH_4_ (aq) + CoM‑S‑S-CoB | -13.95 |
| Eq. 8 | CO_2_ (aq) + 2 Fd (red) + 2 F_420_ (red) + CoM-SH + CoB-SH → CH_4_ (aq) + 2 Fd (ox) + 2 F_420_ (ox) + CoM-SS-CoB + 2H_2_O | -62.11 |
| Eq. 9 | NADP (red) → NADP (ox) + 2H^+^ + 2e^-^ | 14 |
| Eq. 10 | 2Fd (red) → 2Fd (ox) + 2H^+^ + 2e^-^ | **-**16 |
| Eq. 11 | F_420_ (red) → F_420_ (ox) + 2H^+^ + 2e^-^ | 11 |
| Eq. 12 | CO_2_ (aq)+ 2 NADP (red) + F_420_ (red) + CoM-SH + CoB-SH → CH_4_ (aq) + 2 NADP (ox) + F_420_ (ox) + CoM-SS-CoB + 2H_2_O | -29.11 |

F_420_, coenzyme F_420_; Fd, ferredoxin; H_4_MPT, tetrahydrosarcinapterin; HS-CoB, coenzyme B; HS-CoM, coenzyme M; MFR, methanofuran;

MP, methanophenazine.


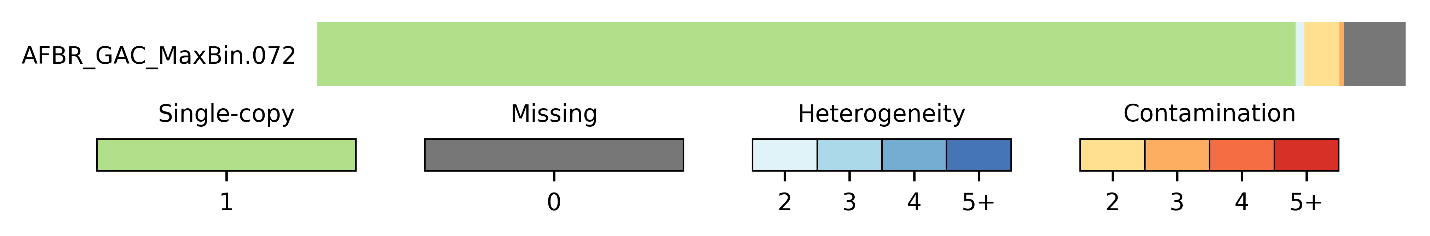


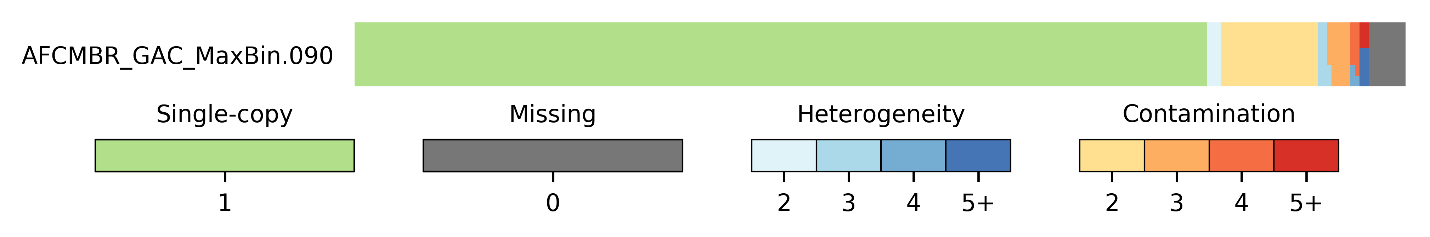


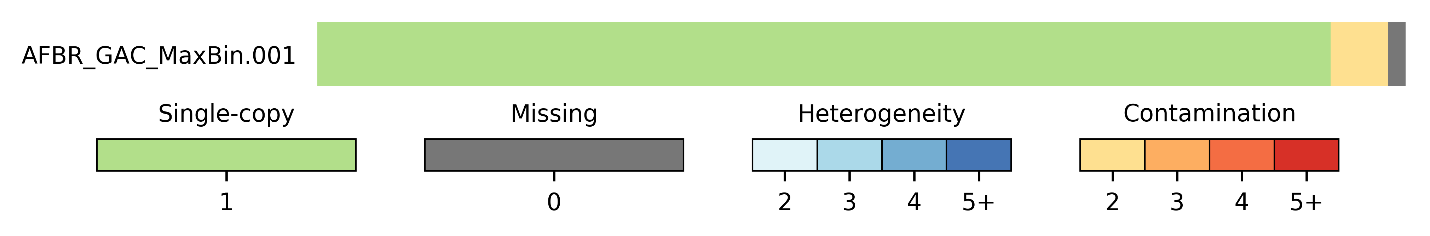


**Figure S1.** CheckM plot for the quality assessment of the genomes bins for further annotation and analysis.


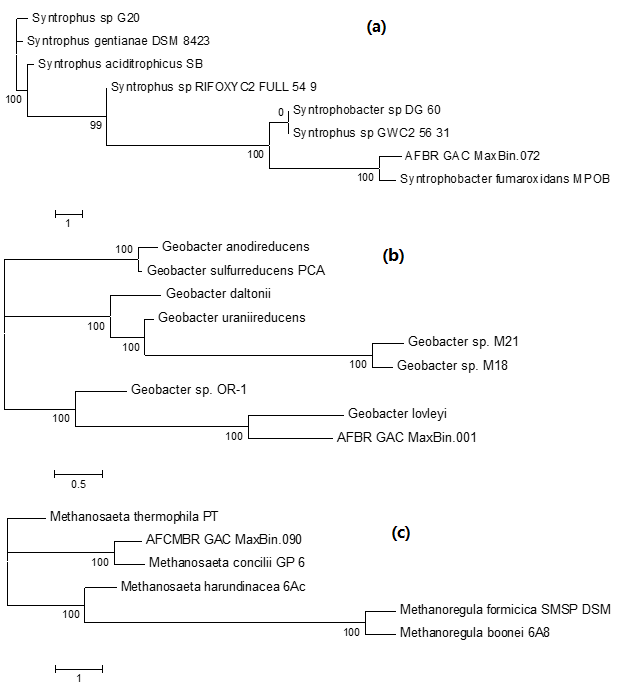


**Figure S2.** Phylogenetic tree of genome bins closely related to (a). *Syntrophobacter fumaroxidans*, (b). *Geobacter lovleyi* and (c). *Methanothrix concilii* using PhyloPhlAn.

**References**

1. M. Aslam, P. Yang, P. H. Lee and J. Kim, *J Membrane Sci*, 2018, **553**, 200-208.

2. R. K. Thauer, A. K. Kaster, H. Seedorf, W. Buckel and R. Hedderich, *Nat Rev Microbiol*, 2008, **6**, 579-591.
